# Analysis of the conditions for applying BRCA genetic testing to women with breast cancer using the Japanese HBOC consortium and the Japanese organization of hereditary breast and ovarian cancer (JOHBOC) registry project database

**DOI:** 10.1007/s12282-025-01704-8

**Published:** 2025-05-05

**Authors:** Masato Takahashi, Yuko Minoura, Hiroki Den, Tadashi Nomizu, Takanori Ishida, Hiraku Kumamaru, Masami Arai, Seigo Nakamura

**Affiliations:** 1 Department of Breast Surgery, Hokkaido University Hospital, Sapporo, Japan; 2 Cancer Institute Hospital, Japanese Foundation for Cancer Research, Tokyo, Japan; 3 Department of Hygiene, Public Health, and Preventative Medicine, School of Medicine, Showa University, Tokyo, Japan; 4 Department of Surgery, Hoshi General Hospital, Koriyama, Japan; 5 Breast and Endocrine Surgical Oncology, Tohoku University Graduate School of Medicine, Sendai, Japan; 6 Department of Healthcare Quality Assessment, The University of Tokyo Graduate School of Medicine, Tokyo, Japan; 7 Department of Clinical Genetics, Graduate School of Medicine, Juntendo University, Tokyo, Japan; 8 Institute for Clinical Genetics and Genomics, Showa University, Tokyo, Japan

**Keywords:** *BRCA1/2*, Genetic testing, Breast cancer, Conditions, Japan

## Abstract

**Background:**

Considering past research in Europe and the USA, the conditions for medical insurance coverage of *BRCA1/2* genetic testing (GT) in Japan have been established as follows: 1. Breast cancer onset at 45 years or younger age; 2. Triple-negative breast cancer (TNBC) onset at 60 years or younger age; 3. Onset of two or more primary breast cancers; 4. Family history of breast cancer, ovarian cancer, or pancreatic cancer up to the third degree; 5. Male breast cancer, 6. Ovarian, fallopian, or peritoneal cancers. However, data to determine the importance and extent of each factor in the current conditions are insufficient. Consequently, this study aimed to assess the validity of insurance coverage conditions in Japan, elucidate the relationship between these conditions, and explore the possibility of proposing new indicators.

**Methods:**

A total of 5987 breast cancer patients were enrolled from 92 centers participating in the HBOC consortium and the JOHBOC registry project. Of these, 5904 patients were analyzed after excluding 48 male breast cancer patients due to insufficient numbers for analysis and 35 patients whose age at breast cancer onset was unknown or unregistered. We compared 1,091 cases in which pathogenic variants (PVs) (*BRCA1*(B1s): 543, *BRCA2*(B2s): 548) were detected with 4580 cases in which no variants (non-Vs) were detected. Variants of uncertain significance (VUS: 233 cases) were not classified as either PVs or non-Vs for subsequent analysis. We investigated the validity of each condition under which an HBOC diagnosis could be considered for medical insurance coverage.

**Results:**

Regardless of the insurance coverage conditions, the detection rate of pathogenic variants (DRPV) of all analyzed cases was 19.2%. The DRPV under the insurance coverage conditions for GT—‘Age of breast cancer onset ≤ 45 years,’ ‘TNBC onset at ≤ 60 years,’ ‘ ≥ 2 primary breast cancers,’ ‘Patients with breast cancer concurrent with ovarian cancer,’ and ‘ ≥ 1 family history of breast or ovarian cancer up to the third degree’—was 25.4%, 31.6%, 24.6%, 48.8%, and 25.6%, respectively. Those within the insurance coverage group showed a pathogenic variant detection rate of 21.1%, compared to only 5.6% outside of the coverage. Our analysis indicates that medical insurance coverage conditions effectively identify candidates for GT. Furthermore, when examining the number of conditions met and the positivity rate, the positivity rate was 11.2%, with only one condition met. This rate increases exponentially as more conditions are met, underscoring the importance of multiple matching conditions. Additionally, those with comorbid ovarian cancer or a family history of ovarian cancer are more likely to have a pathogenic variant. Additionally, we reevaluated cases who did not meet the medical insurance conditions. TNBC occurrence was significantly associated with B1s, even when restricted to onset age ≥ 61 years. Familial history of prostate cancer also significantly associated with B2s.

**Conclusion:**

This study determined that the Japanese medical insurance coverage conditions effectively identified candidates eligible for GT. Consequently, it is imperative to disseminate information to all patients with breast cancer covered by insurance, emphasizing the opportunity for GT, particularly if they have ovarian cancer complications or a family history of ovarian cancer.

## Introduction

Hereditary breast and ovarian cancer (HBOC) predispose individuals to cancer because of germline pathogenic variants in *BRCA1* or *BRCA2* [1]. Since they are genetically more susceptible to developing breast and/or ovarian cancer, early cancer detection, risk-reducing surgery, and/or chemoprevention are necessary to improve survival rates [2–4].

While genetic analyses were initially conducted in Europe and the USA, it has become evident that HBOC exists in Asians at a rate similar to that in Europeans and Americans [5, 6]. PARP inhibitors are recommended for breast cancer recurrence and perioperative treatment, contributing to the rapid advancement of HBOC research [7–9].

Considering past research results from Europe and the USA, as well as the current situation in Japan, the conditions for medical insurance coverage of *BRCA1/2* genetic testing (GT) in Japan were established to include one or more of the following:

- 1.Breast cancer onset at 45 years old or younger
- 2.Triple-negative breast cancer (TNBC) onset at 60 years old or younger
- 3.Onset of 2 or more primary breast cancers
- 4.Family history of breast, ovarian, or pancreatic cancer up to third-degree
- 5.Male breast cancer
- 6.Having ovarian cancer, fallopian tube cancer, or peritoneal cancer

Consequently, a significant number of tests have been conducted in Japan, and the results have been collected from the Japan Organization of the Breast and Ovarian Cancer (JOHBOC) database.

In contrast, the National Comprehensive Cancer Network (NCCN) has established guidelines that serve as a reference for individuals requiring GT [10]. However, disparities exist between these guidelines and the conditions for medical insurance coverage in Japan, and there are insufficient data to determine the importance and extent of each factor in the current situation.

Consequently, we analyzed the positivity rate of pathogenic variants based on the risk factors for GTs in Japan using data from the JOHBOC database. Our aim was to assess the validity of insurance coverage conditions established in Japan, elucidate the relationship between these conditions and pathogenicity, and explore the possibility of proposing new indicators.

## Patients and methods

**Fig. 1:**
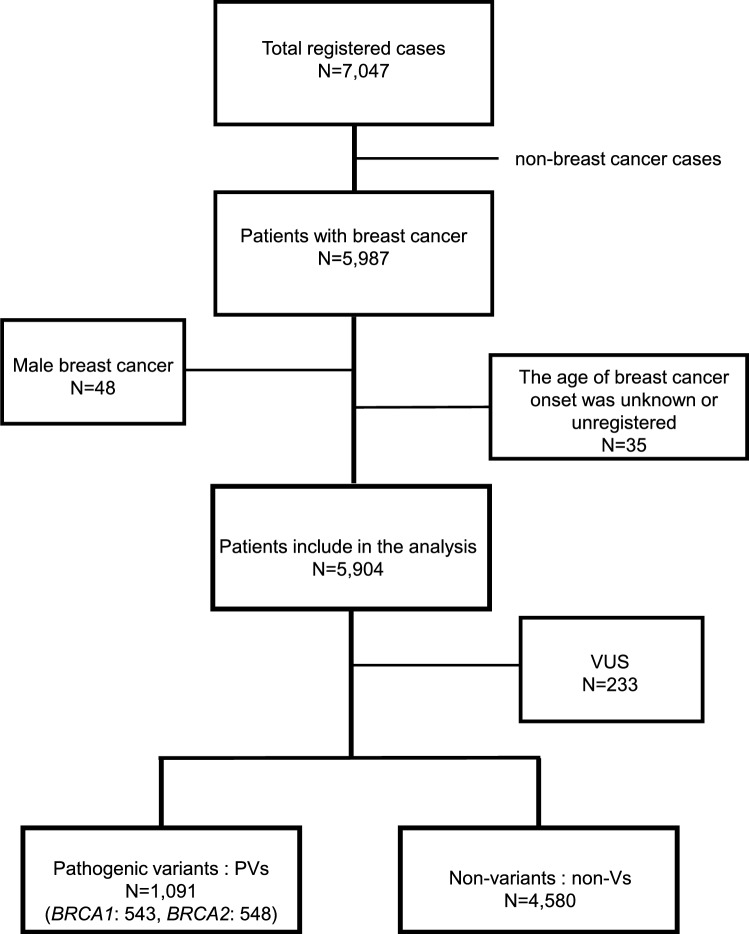
The participants of this study. The JOHBOC database contained a total of 7047 cases, of which 5987 were breast cancer patients. After excluding cases of male breast cancer, which were too few for meaningful analysis, and cases with unknown age at onset, 5,904 cases were included in the analysis. Among them, 233 cases of VUS were excluded from the analysis. Finaly 1091 cases had a pathogenic variant in *BRCA1* or *BRCA2*. Additionally, among the cases without a pathogenic variant, 4580 cases were used for analysis. *Non-Vs* non-variants, *VUS* variants of uncertain significance

From 2015 to 2020, 5987 patients with breast cancer among 7047 *BRCA1/2* GT patients were enrolled from 92 centers participating in the HBOC consortium and the JOHBOC registry project. Of these, 5904 patients were analyzed after excluding 48 male breast cancer patients due to insufficient numbers for analysis and 35 patients whose age at breast cancer onset was unknown or unregistered. We compared 1,091 cases in which pathogenic variants were detected (PVs) (*BRCA1*(B1 s): 543, *BRCA2*(B2 s): 548) with 4,580 cases in which no variants (non-Vs) were detected. Variants of uncertain significance (VUS: 233 cases) were not classified as either PVs or non-Vs for subsequent analysis (Fig. 1). We investigated the validity of each condition under which an HBOC diagnosis could be considered for medical insurance coverage.

For each condition, we calculated the percentage of patients and median age of breast cancer onset in each *BRCA1*, *BRCA2*, and non-Vs group. In the family history section, we evaluated the family histories of breast and ovarian cancers separately to assess the differences by genotype. We calculated the percentage of patients with a family history from the total number of patients in each group by the degree of kinship up to the third degree (duplicates were available). The current medical insurance coverage conditions for *BRCA1/2* GT in Japan include a family history of pancreatic cancer. However, because the insurance coverage conditions during the analyzed registration period did not include a family history of pancreatic cancer, only a family history of breast and/or ovarian cancer was included in this analysis. We also calculated the detection rate of pathogenic variants in individuals who met each condition, the percentage of patients who met each condition only, the number of applicable conditions, and the positive rate. Additionally, we reevaluated cases who did not meet the medical insurance conditions for the following factors: applicable to the condition for companion diagnosis of PARP inhibitor only, triple-negative breast cancer first onset at ≥ 61y, breast cancer onset at ≥ 46y without TNBC, and family history of pancreatic or prostate cancers.

### Statistical analysis

All statistical analyses were conducted using the R software version 4.2.2 (R Foundation for Statistical Computing, Vienna, Austria). Fisher's exact test was used to compare categorical data.

The Kruskal–Wallis test was utilized to compare the medians of multiple groups.

To compare proportions between groups, a test for equality of proportions was applied. For multiple comparisons, the Bonferroni correction method was employed to adjust the p-values obtained from the Kruskal–Wallis test and the test for equality of proportions. Logistic regression analysis was performed with the presence of pathogenic variants serving as the dependent variable and medical insurance coverage conditions serving as independent variables.

Additionally, three separate logistic regression models were constructed: (1) adding TNBC first onset at age ≥ 61 years as an independent variable, (2) adding breast cancer onset at age ≥ 46 years without TNBC, and (3) simultaneously adding family history of pancreatic cancer and family history of prostate cancer as independent variables. The condition for companion diagnosis of PARP inhibitors"refers to the results of tests conducted to determine the eligibility for pharmacological therapy. This differs from the objective of exploring conditions under which GT should be performed beyond the current insurance coverage. Therefore, logistic analysis was not conducted for this item.

A significance level of p < 0.05 was used to determine statistical significance in all analyses.

## Results

**Fig. 2:**
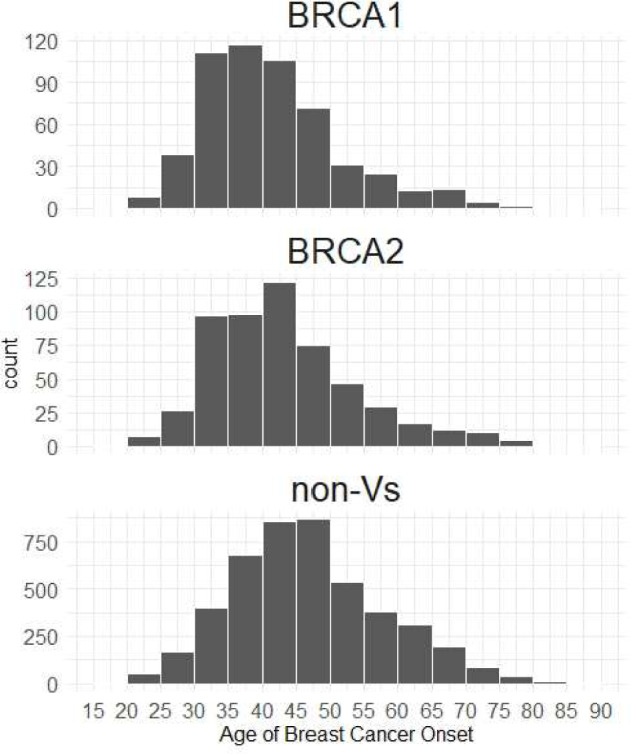
The peak age of breast cancer onset. **a**. *BRCA1* pathogenic variants **b** *BRCA2* pathogenic variants **c** non-variants. The length of the bar graph indicates the number of individuals with breast cancer onset. The histogram suggests that cases with pathogenic variants tend to develop breast cancer at a younger age compared to non-Vs. Additionally, *BRCA1* cases tend to have an even earlier onset compared to *BRCA2* cases. Due to the small number of VUS cases, analysis is challenging

Of 5904 female breast cancer patients analyzed, variants of uncertain significance (VUS: 233 cases) were not classified as either PVs or non-Vs. These cases with VUS were excluded from subsequent analysis. Pathogenic variants of *BRCA1* were detected in 543 cases (B1 s), and *BRCA2* variants were detected in 548 cases (B2 s). While 4,580 cases had no variants at all (non-Vs). The positivity rate of PVs for non-Vs was 19.2% (1,091/4,580) in patients with breast cancer. Note that in seven cases, PVs were detected in both *BRCA1* and *BRCA2,* these seven cases were analyzed as B1 s for simplicity. 1,052 (B1 s: 98.9% [537/543], B2 s: 94.0% [515/548]) PVs and 3,923 (85.7% [3923/4580]) in non-Vs were applicable for any of the medical insurance coverage conditions for HBOC diagnosis, with particularly high rates in B1 s compared to other groups (B1 s vs. B2 s: p < 0.001, B1 s vs. non-Vs: p < 0.001). The median ages at breast cancer onset was 40/42/46 years for B1 s/B2 s/non-Vs, respectively. PVs developed breast cancer at a significantly younger than non-Vs (B1 s vs. non-Vs: p < 0.001, B2 s vs. non-Vs: p < 0.001). The peak age of breast cancer onset was 36–40 years in B1 s, 41–45 years in B2 s, and 46–50 years in non-Vs (Fig. 2).

### 3–1. The applicability rate to medical insurance coverage conditions of HBOC diagnosis (MICC) (Table 1)

**Table 1:** The applicability rate to medical insurance coverage conditions of HBOC diagnosis; n (%)

	Pathogenic Variant (n = 1,091)		Non- Variants (n = 4,580) (%)	DRPV (%)	VUS (n = 233) (%)	P-value^#^
	*BRCA1* (n = 543) (%)	*BRCA2* (n = 548) (%)				
Age of breast cancer onset ≤ 45 years	381 (70.2)	352 (64.2)	2,158 (47.1)	25.4	126 (54.1)	P < 0.001
TNBC onset at ≤ 60 years	331 (61.0)	68 (12.4)	864 (18.9)	31.6	53 (22.7)	P < 0.001
≥ 2 primary breast cancer	146 (26.9)	130 (23.7)	845 (18.4)	24.6	42 (18.0)	P < 0.001
Patients with breast cancer concurrent ovarian cancer	78 (14.4)	28 (5.1)	111 (2.4)	48.8	8 (3.4)	P < 0.001
≥ 1 family history of breast or ovarian cancer up to third-degree	413 (76.1)	394 (71.9)	2351 (51.3)	25.6	132 (56.7)	P < 0.001

*DRPV* the detection rate of pathogenic variants, *VUS* variants of uncertain significance, *TNBC* triple-negative breast cancer

^#^P values were calculated excluding VUS

#### 3–1-1. Breast cancer onset at ≤ 45 y

**Fig. 3:**
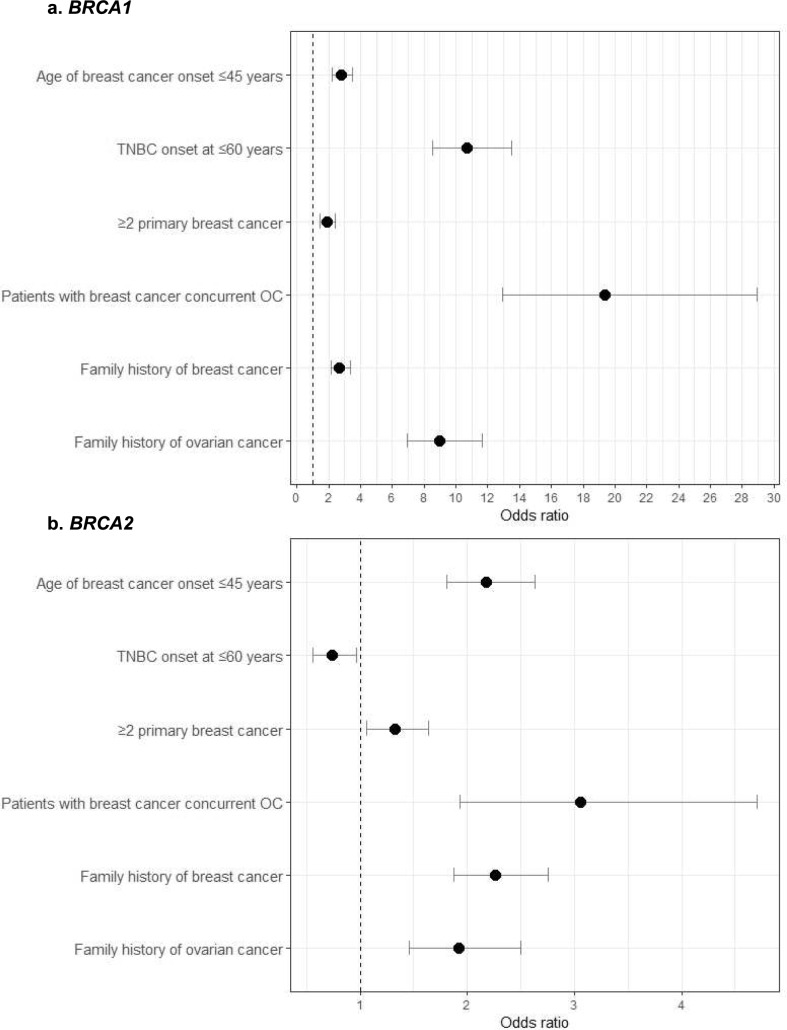
The odds ratio of the detection rate of pathogenic variants for each insurance coverage condition **a** *BRCA1*pathogenic variants vs. non variants; **b** *BRCA2* pathogenic variants vs. non- variants. The odds ratios for pathogenic variants detection were calculated separately for *BRCA1* and *BRCA2* according to each factor related to insurance coverage. Among patients with breast cancer concurrent ovarian cancer, both B1 s and B2 s exhibited the highest odds ratios. However, the odds ratio for B1 s was more than six times higher than that for B2 s. TNBC onset at ≤ 60y was associated with a high odds ratio for B1 s, whereas no significant increase was observed for B2 s. B1 s: BRCA1 pathogenic variants, B2 s: BRCA2 pathogenic variants TNBC: triple-negative breast cancer. odds ratio, the bar length: 95% Confidence Interval

The applicability rate was 70.2% in B1 s (381/543), 64.2% in B2 s (352/548), and 47.1% in non-Vs (2,158/4,580) (p < 0.0001). The detection rate of pathogenic variants in individuals who met this condition (DRPV) was 25.4%. Of all participants, 718 met only this condition, of which 9.7% (70 cases: 13 in B1 s and 57 in B2 s) had pathogenic variants. The odds ratio for the detection rate of breast cancer onset at ≤ 45 years was 2.79 (95% CI: 2.22–3.52) for B1 s and 2.18 (95% CI: 1.81–2.64) for B2 s (Fig. 3) (Table 2).

**Table 2:** The relative risk of pathogenic variants for each medical insurance coverage conditions

	*BRCA1* pathogenic variants		*BRCA2* pathogenic variants	
	Odds ratio	95% CI	Odds ratio	95%CI
Age of breast cancer onset ≤ 45 years	2.79	2.22–3.52	2.18	1.81–2.64
TNBC onset at ≤ 60 years	10.68	8.50–13.51	0.74	0.56–0.96
≥ 2 primary breast cancer	1.89	1.47–2.42	1.32	1.06–1.64
Patients with breast cancer concurrent ovarian cancer	19.33	12.91–28.95	3.06	1.93–4.71
≥ 1 family history of breast cancer up to third-degree	2.66	2.14–3.34	2.27	1.87–2.75
≥ 1 family history of ovarian cancer up to third-degree	8.96	6.91–11.64	1.92	1.46–2.50

*CI* Confidence interval, *TNBC* triple-negative breast cancer

#### 3–1–2. Triple-negative breast cancer (TNBC) onset at ≤ 60y.

The applicability rate was 61.0% in B1 s (331/543), 12.4% in B2 s (68/548), and 18.9% in non-Vs (864/4,580) (p < 0.0001). B1 s had significantly higher applicability rates (61.0%) than the other groups (B1 s vs. B2 s, p < 0.001; B1 s vs. non-Vs, p < 0.001). Of all the participants, 256 met this condition exclusively, of which 8.2% (21 cases; 14 in B1 s and 7 in B2 s) were detected with pathogenic variants. The odds ratio for the detection rate of TNBC onset at ≤ 60y was 10.68 (95% CI: 8.50–13.51) for B1 s and 0.74 (95% CI: 0.56–0.96) for B2 s. This condition indicated a significantly higher risk of detecting B1 s, while the risk of detecting B2 s was lower.

#### 3–1–3. ≥ 2 primary breast cancer

The applicability rate was 26.9% in B1 s (146/543), 23.7% in B2 s (130/548), and 18.4% in non-Vs (845/4,580) (p < 0.0001). The DRPV was 24.6%. Of all participants, 177 met this condition exclusively, of which 5.6% (10 cases: 4 in B1 s and 6 in B2 s) were detected with pathogenic variants, and the median age of first breast cancer onset was 38/42/46 years for B1 s/B2 s/non-Vs, respectively (p < 0.001). The median ages at second breast cancer onset in patients who developed metachronously breast cancer were 46/49/50 years, respectively (p < 0.001). The median time to second cancer onset was 6/4/2.5 years (p < 0.001).

#### 3–1–4. Patients with breast cancer concurrent ovarian, fallopian, or peritoneal cancer (OC)

The applicability rate was 14.4% for B1 s (78/543), 5.1% for B2 s (28/548), and 2.4% for non-Vs (111/4,580) (p < 0.001). B1 s had significantly higher applicable rates than the other groups (B1 s vs B2 s: p < 0.001, B1 s vs non-Vs: p < 0.001). The DRPV was 48.8%. Of all the participants, 59 met this condition exclusively, of which 25.4% (15 cases: 8 in B1 s and 7 in B2 s) were detected with pathogenic variants. The odds ratio for the detection rate of ‘Patients with breast cancer concurrent ovarian, fallopian, or peritoneal cancer (OC)’ was 19.33 (95% CI: 12.91–28.95) for B1 s and 3.06 (95% CI: 1.93–4.71) for B2 s. The risk of detecting PV under this condition was significantly high for both B1 s and B2 s, with a particularly pronounced increase in risk for B1 s. The median age of breast cancer onset in this condition was 46/54/54 years for B1 s/B2 s/non-Vs. B1 s onset younger than those in the other groups (B1 s vs. B2 s, p = 0.023; B1 s vs. non-Vs, p < 0.001). The median age at OC onset was 50.5/60/54.5 years, with B1 s onset being younger than B2 s (p < 0.001). In all groups, the proportion of those who first developed breast cancer was higher than that of those with ovarian cancer (breast cancer, 57.5%; ovarian cancer, 34.6%; unknown, 7.9%).

#### 3–1–5. ≥ 1 family history of breast or ovarian cancer up to third-degree

The applicability rate was 76.1% in B1 s (413/543), 71.9% in B2 s (394/548), and 51.3% in non-Vs (2,351/4,580) (p < 0.001). The DRPV was 25.6%. Of all participants, 988 met only this condition, of which 13.3% (131 cases: 41 in B1 s and 90 in B2 s) were detected with pathogenic variants. B2 s had a higher rate of applying only this condition than B1 s (p < 0.001). The odds ratio was analyzed separately based on the presence or absence of a family history of breast cancer and ovarian cancer. The odds ratio for the detection rate of ‘ ≥ 1 family history of breast cancer’ was 2.66 (95% CI: 2.13–3.34) for B1 s and 2.27 (95% CI: 1.87–2.75) for B2 s. And the odds ratio for the detection rate of ‘ ≥ 1 family history of ovarian cancer’ was 8.96 (95% CI: 6.91–11.64) for B1 s and 1.92 (95% CI: 1.46–2.50) for B2 s. On all conditions, the risk of detecting PV was significantly high; however, the risk of detecting B1 s was particularly elevated in cases with a family history of ovarian cancer. Table.3 shows the proportion of individuals who had ≥ 1 family history of breast cancer and ovarian cancer up to 3rd degree, respectively. In both the family histories of breast and ovarian cancer, PVs had a significantly higher rate of family history than non-Vs, excluding the family history of ovarian cancer in B2 s vs. non-Vs (2nd degree).

**Table 3:** Family history of breast or ovarian cancer up to third-degree

Family history	Degree of kinship	*BRCA1* (n = 543)		*BRCA2* (n = 548)		non-Vs; % (n = 4,580)
		N	P-value (vs non-Vs)	N	P-value (vs non-Vs)	
Breast cancer	1 st	229	< 0.001	261	< 0.001	1302
	2nd	194	< 0.001	207	< 0.001	1071
	3rd	81	0.001	82	0.001	449
Ovarian cancer	1 st	123	< 0.001	46	< 0.001	175
	2nd	100	< 0.001	29	0.20	165
	3rd	22	< 0.001	11	0.02	35

*non-V*s non-variants

In all groups, especially the PVs, there was a tendency for family history to decrease with increasing distance from kinship.

#### 3–1–6. The number of applicable conditions and positive rate

The number of applicable items for each group and the positivity rates for the above five conditions excluding male breast cancer are shown in Table 4. Those within the insurance coverage group showed a pathogenic variant detection rate of 21.1% (1052/4975), compared to only 5.6% (39/697) outside of the coverage. Our analysis indicates that medical insurance coverage conditions effectively identify candidates for GT. The median numbers of applicable conditions were 2.0 in the PVs and 1.0 in n non-Vs. Regarding the positive rate, 11.2% of cases with only one of the five conditions met had a pathogenic variant. This increased to 21.5% when two conditions were met. This rate increased exponentially as more conditions were met, underscoring the importance of multiple matching conditions.

**Table 4:** The number of applicable conditions and positive rate

The number of applicable conditions	0	1	2	3	4	5
Pathogenic Variant (n = 1091)	39	247	428	293	81	3
Non-Variant (n = 4580)	657	1951	1567	376	29	0
Positive rate (%)	5.6	11.2	21.5	43.8	73.6	100

### 3–2. Evaluation of cases that did not meet the above conditions (Table 5)

**Table 5:** Evaluation of cases that did not meet the insurance coverage conditions

	Pathogenic Variant (n = 1091)		Non- Variants (n = 4580) (%)	DRPV (%)	BRCA1		BRCA2	
	*BRCA1* (n = 543) (%)	*BRCA2* (n = 548) (%)			Odds ratio	95%CI	Odds ratio	95%CI
Companion diagnostics of PARP inhibitor	54 (9.9)	92 (16.8)	865 (18.9)	14.4	NA	NA	NA	NA
TNBC onset at ≥ 61 years	33 (6.1)	18 (3.3)	227 (5.0)	18.3	7.52	4.56–12.16	1.19	0.69–1.93
Breast cancer onset at ≥ 46y without TNBC	68 (12.5)	156 (28.5)	1830 (40.0)	10.9	0.72	0.47–1.11	0.72	0.48–1.09
Family history of pancreatic cancers	34 (6.3)	37 (6.8)	214 (4.7)	24.9	1.19	0.74–1.84	1.27	0.87–1.82
Family history of prostate cancers	77 (14.2)	122 (22.3)	619 (13.5)	24.3	0.76	0.52–1.10	1.68	1.30–2.14

*DRPV* the detection rate of pathogenic variants, *CI* Confidence interval, *NA* no analysis, *TNBC* triple-negative breast cancer

#### 3–2-1. Applicable to the condition for companion diagnosis of PARP inhibitor (CDx)

Although this may not strictly fall within the scope of insurance coverage conditions for testing, some cases were tested as companion diagnostics. Therefore, the results are presented here. The number of registered patients who underwent GT as CDx was 1011, among them, that of which did not meet the MICC conditions was 304. Pathogenic variants were detected in 14.4% of the cases (146 cases PVs: 54 B1 s and 92 B2 s 856 non-Vs). B2 s had significantly higher detection rates than B1 s. (p = 0.025).

#### 3–2-2. Triple-negative breast cancer (TNBC) first onset at ≥ 61y

The registered patients who developed TNBC as their first breast cancer ≥ 61y and did not meet MICC were 121 cases. Pathogenic variants were detected in 18.3% of the cases (51 cases PVs: 33 B1 s and 18 B2 s; 227 non-Vs). The median age at breast cancer onset was 64/67.5/65 years, for B1 s/B2 s/non-Vs, respectively (p = 0.2). The odds ratio for the detection rate of ‘Triple-negative breast cancer (TNBC) first onset at ≥ 61y’ was 7.52 (95% CI: 4.56–12.16) for B1 s and 1.18 (95% CI: 0.69–1.93) for B2 s. TNBC occurrence was significantly associated with B1 s, even when restricted to onset age ≥ 61 years.

#### 3–2–3. Breast cancer onset at ≥ 46y without TNBC

The registered patients who developed breast cancer onset at ≥ 46y without TNBC and did not meet MICC were 575 cases. Pathogenic variants were detected in 10.9% of the cases (224 cases PVs: 68 B1 s and 156 B2 s; 1,830 non-Vs). B2 s had significantly higher detection rates than B1 s (p < 0.001). The median ages at breast cancer onset were 52/52/53 years, for B1 s/B2 s/non-Vs, respectively (p = 0.53). The odds ratio for the detection rate of ‘Breast cancer onset at ≥ 46y without TNBC’ was 0.72 (95% CI: 0.47–1.11) for B1 s and 0.72 (95% CI: 0.48–1.09) for B2 s.

#### 3–2–4. Family history of pancreatic or prostate cancers

The registered patients with ≥ 1 family history of pancreatic or prostate cancer as HBOC-related cancers and did not meet MICC were 59 cases. A family history of pancreatic cancer is currently covered by insurance in Japan. However, during the period analyzed in this study (2015–2020), it was not covered by insurance; therefore, we report the results of case accumulation here. Pathogenic variants were detected in 24.9% of the cases (71 cases PVs: 34 B1 s and 37 B2 s; 214 non-Vs) with family history of pancreatic cancer. The odds ratio for the detection rate of ‘ ≥ 1 family history of pancreas cancer’ was 1.18 (95% CI: 0.74–1.83) for B1 s and 1.27 (95% CI: 0.87–1.82) for B2 s. Pathogenic variants were detected in 24.3% of the cases (199 cases PVs: 77 B1 s and 122 B2 s; 619 non-Vs) with family history of prostate cancer. The odds ratio for the detection rate of ‘ ≥ 1 family history of prostate cancer’ was 0.76 (95% CI: 0.51–1.10) for B1 s and 1.67 (95% CI: 1.29–2.14) for B2 s. In B2 s, family histories of prostate cancer were significantly associated with pathogenic variants.

## Discussion

Approximately 100,000 new cases of breast cancer are reported annually in Japan and approximately 5% of these cases are attributable to HBOC [5]. JOHBOC has accumulated GTs data for nearly all individuals who have undergone GTs, with a special focus on collecting data from all JOHBOC participating facilities until 2020, regardless of whether a pathogenic variant was detected. This comprehensive database provides essential data for assessing the status of GTs in HBOC in Japan.

Testing for HBOC can significantly expand treatment options, including the inclusion of risk-reducing surgical procedures and indications for treatment with PARP inhibitors [2–4]. However, it is also important to consider drawbacks, such as the cost of testing and the potential harm associated with genetic information [11]. Therefore, it is crucial to make informed decisions about which patients with breast cancer should undergo GT in Japan. When determining the medical insurance coverage for this test, the decision was initially based on past reports of HBOC risks in Europe and the USA [12, 13]. However, as no studies have been conducted using actual cases, we aimed to assess the validity of these conditions and determine the extent of their applicability in our context.

Our analysis indicates that the medical insurance coverage conditions are effective in identifying candidates for GT. Those within the insurance coverage group showed a pathogenic variant detection rate of 21.1%, compared to only 5.6% outside of the coverage. This fourfold increase underlines the appropriateness of these conditions.

Each insurance coverage condition was analyzed individually. Notably, individuals with B1 s and B2 s develop breast cancer at a significantly younger age than those without these variants. Furthermore, an insurance coverage age limit of 45 years or younger bridges the gap between the median age of onset for individuals with and without these variants. The results of the analysis of the median age of onset for PVs and non-Vs cases appear to validate this age limit as a condition for insurance coverage.

This analysis revealed that'triple-negative breast cancer onset at 60 years old or younger'is a reasonable condition for insurance coverage from PV rate. However, patients with triple-negative breast cancer with first onset at 61 years of age or older are also likely to have germline variants in *BRCA1*, indicating that the risk is not low. The NCCN and other guidelines do not specifically limit testing for triple-negative breast cancer based on the age of GT [10, 14]. Therefore, there is a need to discuss potential changes to these conditions in Japan.

Regarding the presence of two or more primary breast cancers, some cases were associated with PV. However, when this condition was the sole condition, only 5.6% of the cases were identified as PV, indicating that its significance differed from that of the other conditions. It is believed that the importance of this factor increases when it is combined with other factors. Due to the limited number of analyses concerning male breast cancer, its relationship with PV remains a topic for future research.

Cases of breast cancer co-occurring with ovarian, fallopian tube, or peritoneal cancers are quite common (OC), suggesting that BRCA testing should be considered based solely on this condition. Interestingly, it can be inferred that cases with a late onset of OC and those with breast cancer are likely to have *BRCA2* variants. Furthermore, because breast cancer often occurs before OC, performing a BRCA test at the onset of breast cancer can reduce the risk of OC. If hereditary breast and ovarian cancer (HBOC) is confirmed, risk-reducing salpingo-oophorectomy can be a life-saving intervention [3, 4, 15]. This approach was highly beneficial.

Furthermore, when examining the number of cases covered by insurance, the number of conditions met, and the positivity rate for female patients, it was evident that with only one condition met, the positivity rate was 11.2%. In addition, the positivity rate increased exponentially as more conditions were met. This observation underscores the importance of using multiple matching conditions.

The major distinction when comparing *BRCA1* and *BRCA2* lies in the condition of TNBC ≤ 60 years, where the odds ratio for variants in *BRCA1* was over 10, while for *BRCA2*, it was actually less than 1. In cases of ovarian cancer complications, even *BRCA2* exhibit sufficiently high odds ratio of 3.06, but *BRCA1* stood out with an odds ratio of 19.33, indicating a notably high probability. Additionally, when considering a family history of ovarian cancer, *BRCA1* showed a high odds ratios of 8.96, whereas *BRCA2* did not exhibit such a significance compared to *BRCA1*.

Therefore, when taking into account these three factors— personal history of ovarian cancer, family history of ovarian cancer, and TNBC ≤ 60 years — they are particularly important for cases with *BRCA1* pathogenic variants. Furthermore, TNBC are at a higher risk of being associated with *BRCA1* pathogenic variants, whereas cases with *BRCA2* pathogenic variants are less likely to develop TNBC.

In contrast, a family history of prostate cancer was associated with PV only in *BRCA2*. Based on the fact that overall *BRCA2* is detected at a lower rate than *BRCA1* within the current coverage conditions and at a higher rate in other conditions, it is necessary to discuss the addition of a family history of prostate cancer to MICC that will increase the detection rate of *BRCA2* [16].

This study provides crucial findings that shape the direction of breast cancer treatment. However, it is important to acknowledge certain limitations when interpreting these results. Although the sample size for the analysis was sufficient, GT for *BRCA1* and *BRCA2* was not conducted for all breast cancer cases at the time of the study. Consequently, the frequency of *BRCA1* and *BRCA2* pathogenic variants in untested cases remains unclear. Nonetheless, because we were able to analyze some cases that did not meet the MICC conditions, it is likely that the absence of testing in all cases did not significantly impact the analysis. Moreover, although the current data regarding the frequency of *BRCA1* and *BRCA2* pathogenic variants based on various conditions are valuable, it remains unclear which clinical conditions are associated with pathogenic variants of other genes, such as *PALB2* and *TP53* [2, 5, 17]. This has not been fully elucidated. A comprehensive analysis of these factors is a challenge for future research.

This study determined that the Japanese medical insurance coverage conditions effectively identified candidates eligible for GT. Additionally, the likelihood of a pathogenic variant varies according to these conditions, with patients with ovarian cancer complications and the presence or absence of a family history of ovarian cancer being particularly prone to carrying such variants. Consequently, it is imperative to disseminate information to all patients with breast cancer covered by insurance, emphasizing the opportunity for GT, particularly if they have ovarian cancer complications or a family history of ovarian cancer. It is crucial to clearly communicate the possibility of detection and provide options such as risk-reducing surgery and appropriate drug therapy when a pathogenic variant is identified by testing.

## Data Availability

In principle, access to the JOHBOC dataset is granted through an annual public call for applications. Interested parties are invited to submit proposals during this period, which are then reviewed by both the Registration Committee and the Academic Committee. The dataset is made available only to those applications that are formally approved. This process ensures that the JOHBOC Secretariat maintains full oversight and accountability throughout all stages of data utilization, including the stated purpose, method of use, and any subsequent dissemination through presentations or academic publications.

## References

[1] Kuchenbaecker KB, Hopper JL, Barnes DR, Phillips KA, Mooij TM, Roos-Blom MJ, et al. Risks of Breast, Ovarian, and Contralateral Breast Cancer for BRCA1 and BRCA2 Mutation Carriers. JAMA. 2017;317:2402–16. 28632866 10.1001/jama.2017.7112

[2] Yoshida R. Hereditary breast and ovarian cancer (HBOC): review of its molecular characteristics, screening, treatment, and prognosis. Breast Cancer. 2021;28:1167–80. 32862296 10.1007/s12282-020-01148-2 PMC8514387

[3] Yamauchi H, Nakagawa C, Kobayashi M, Kobayashi Y, Mano T, Nakamura S, et al. Cost-effectiveness of surveillance and prevention strategies in BRCA1/2 mutation carriers. Breast Cancer. 2018;25:141–50. 29019095 10.1007/s12282-017-0803-y

[4] Martelli G, Barretta F, Vernieri C, Folli S, Pruneri G, Segattini S, et al. Prophylactic Salpingo-Oophorectomy and Survival After BRCA1/2 Breast Cancer Resection. JAMA Surg. 2023;158:1275–84. 37792368 10.1001/jamasurg.2023.4770 PMC10551816

[5] Momozawa Y, Iwasaki Y, Parsons MT, Kamatani Y, Takahashi A, Tamura C, et al. Germline pathogenic variants of 11 breast cancer genes in 7,051 Japanese patients and 11,241 controls. Nat Commun. 2018;9:4083. 30287823 10.1038/s41467-018-06581-8 PMC6172276

[6] Enomoto T, Aoki D, Hattori K, Jinushi M, Kigawa J, Takeshima N, et al. The first Japanese nationwide multicenter study of BRCA mutation testing in ovarian cancer: CHARacterizing the cross-sectional approach to Ovarian cancer geneTic TEsting of BRCA (CHARLOTTE). Int J Gynecol Cancer. 2019;29:1043–9. 31263023 10.1136/ijgc-2019-000384

[7] Byrski T, Huzarski T, Dent R, Marczyk E, Jasiowka M, Gronwald J, et al. Pathologic complete response to neoadjuvant cisplatin in BRCA1-positive breast cancer patients. Breast Cancer Res Treat. 2014;147:401–5. 25129345 10.1007/s10549-014-3100-x

[8] Tutt A, Robson M, Garber JE, Domchek SM, Audeh MW, Weitzel JN, et al. Oral poly (ADP-ribose) polymerase inhibitor olaparib in patients with BRCA1 or BRCA2 mutations and advanced breast cancer: a proof-of-concept trial. Lancet. 2010;376:235–44. 20609467 10.1016/S0140-6736(10)60892-6

[9] Tutt ANJ, Garber JE, Kaufman B, Viale G, Fumagalli D, Rastogi P, et al. Adjuvant Olaparib for Patients with BRCA1- or BRCA2-Mutated Breast Cancer. N Engl J Med. 2021;384:2394–405. 34081848 10.1056/NEJMoa2105215 PMC9126186

[10] NCCN.org: Genetic/Familial High-Risk Assessment: Breast, Ovarian, and Pancreatic. NCCN Guideline ver. 3, 2024

[11] Muto K, Nagai A, Ri I, Takashima K, Yoshida S. Is legislation to prevent genetic discrimination necessary in Japan? An overview of the current policies and public attitudes. J Hum Genet. 2023;68:579–85. 37286895 10.1038/s10038-023-01163-z PMC10449614

[12] Müller D, Danner M, Schmutzler R, Engel C, Wassermann K, Stollenwerk B, et al. Economic modeling of risk-adapted screen-and-treat strategies in women at high risk for breast or ovarian cancer. Eur J Health Econ. 2019;20:739–50. 30790097 10.1007/s10198-019-01038-1

[13] Armstrong J, Toscano M, Kotchko N, Friedman S, Schwartz MD, Virgo KS, et al. Utilization and outcomes of BRCA genetic testing and counseling in a national commercially insured population: the ABOUT study. JAMA Oncol. 2015;1:1251–60. 26426480 10.1001/jamaoncol.2015.3048

[14] Sessa C, Balmaña J, Bober SL, Cardoso MJ, Colombo N, Curigliano G, et al. Risk reduction and screening of cancer in hereditary breast– ovarian cancer syndromes: ESMO clinical practice guideline. Ann Oncol. 2023;34:33–47. 36307055 10.1016/j.annonc.2022.10.004

[15] Kotsopoulos J, Gronwald J, Huzarski T, Møller P, Pal T, McCuaig JM, et al. Bilateral oophorectomy and all-cause mortality in women with BRCA1 and BRCA2 sequence variations. JAMA Oncol. 2024;10:484–92. 38421677 10.1001/jamaoncol.2023.6937 PMC10905374

[16] Minoura Y, Takahashi M, Maeda H, Kuwahara S, Tachikawa H, Yamamoto M, et al. Significance of prostate/pancreatic/skin cancer family history for detecting BRCA2 pathogenic variant careers among patients with breast cancer. Breast Cancer. 2022;29:808–13. 35641852 10.1007/s12282-022-01360-2

[17] Easton DF, Pharoah PD, Antoniou AC, Tischkowitz M, Tavtigian SV, Nathanson KL, et al. Gene-panel sequencing and the prediction of breast-cancer risk. N Engl J Med. 2015;372:2243–57. 26014596 10.1056/NEJMsr1501341 PMC4610139

